# Hyposplenism in warm autoimmune hemolytic anemia: The missing link with venous thromboembolism?

**DOI:** 10.1002/jha2.83

**Published:** 2020-08-24

**Authors:** Joaquín Jerez

**Affiliations:** ^1^ Internal Medicine Department School of Medicine Pontificia Universidad Católica de Chile Santiago Chile

A 56‐year‐old man with a previous history of hypothyroidism and obesity presented with progressive dyspnea and palpitations. Hypoxemia was noted in emergency room with a mild tachycardia and tachypnea. Electrocardiogram evidenced sinusal tachycardia.

**FIGURE 1:**
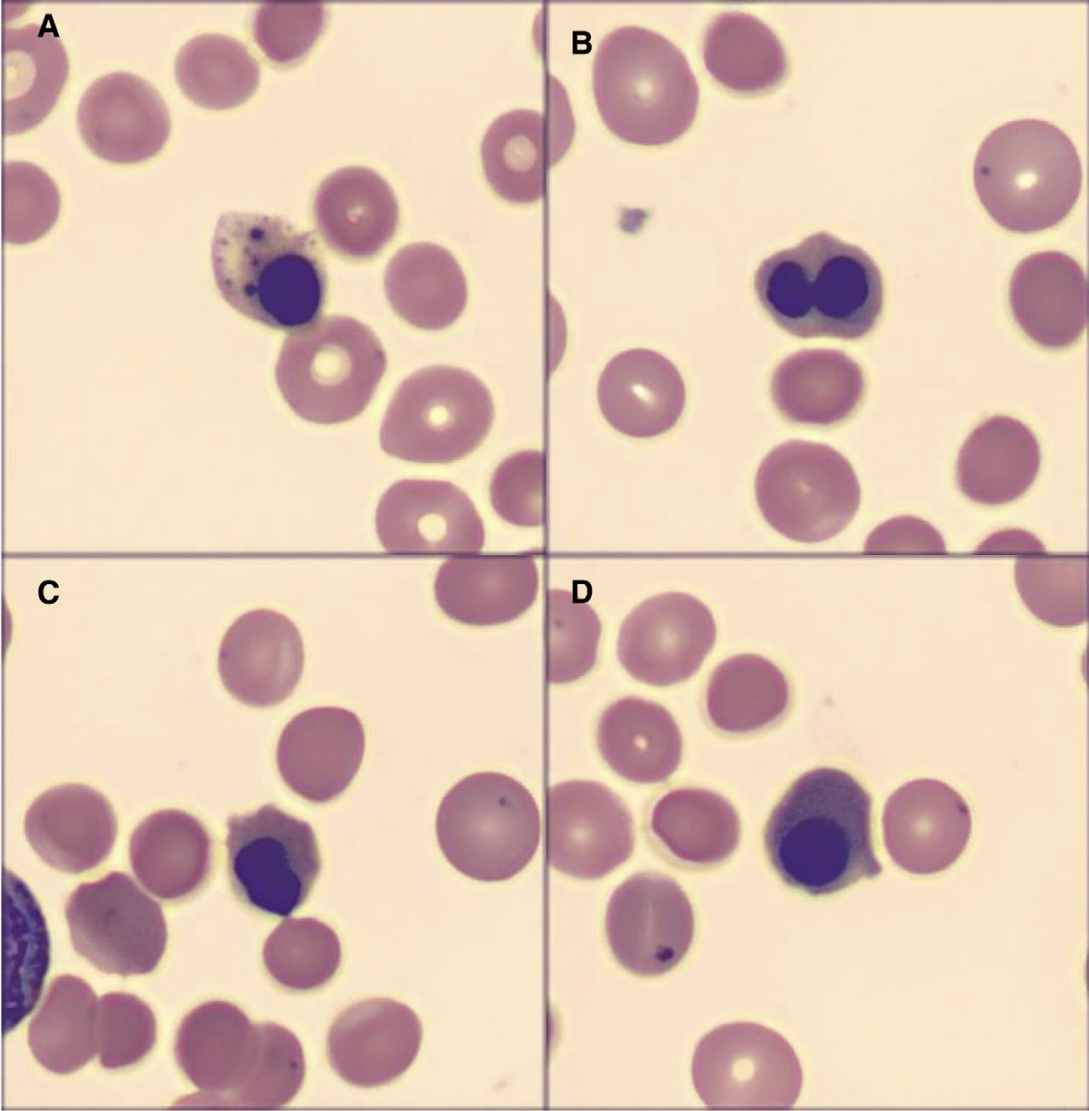
Peripheral blood film; digital microscopy; Cellavision, ×100 objective. A and B, Erythroblasts with dysplastic features and basophilic inclusions. C, Spherocytes. D, Howell‐Jolly bodies

Laboratory workup showed macrocytic anemia (hemoglobin, 8.1 g/dL; MCV, 108 fL), with normal leucocytes and platelets counts.

A direct visualization of blood film was performed: abundant erythroblasts were noted representing 120 for each 100 leucocytes, some with dysplastic features such as basophilic inclusions (Figure 1A [Digital microscopy; Cellavision, ×100 objective]) and binucleated inclusions (Figure 1B). Erythrocytes presented predominantly as spherocytes (Figure 1C) and Howell‐Jolly bodies were noted (Figure 1D). The reticulocyte count was 21%, haptoglobin < 10 mg/dL, LDH 1134 UI/L, and bilirubin 1.72 mg/dL. DAT was strongly positive for IgG, and the diagnosis of warm immune hemolytic anemia was performed. The patient was prescribed prednisone 1 mg/kg daily, and the study for secondary causes was negative.

Patient persisted with dyspnea and tachypnea, and thus CTA was performed. Acute multisegmentary pulmonary thromboembolism was diagnosed, and anticoagulation was started with apixaban.

Initially, hyposplenism was a term used only to describe splenectomized patients [1], but it has since been applied to some systemic illness that cause a detrimental function of spleen, the most recognized ones are celiac sprue and sickle cell disease [2]. Patients with hyposplenism have a major risk not only for severe infections, but also for venous and arterial thrombosis [3]. Hemolytic anemias were proposed as potential cause of hyposplenism, because the spleen macrophages are overloaded trying to remove the damaged erythrocytes, and visualization of diagnostic hallmarks such as Howell‐Jolly bodies was reported recently [4]. Also, the elevated count of erythroblast and the incapacity of macrophages to clear their inclusions can be signs of hyposplenism.

Warm immune hemolytic anemia is associated with an augmented risk of venous thromboembolism, incidence varying between 10 and 20%. Interestingly, 80% of these events presents early in the first 2 months after the diagnosis [5], in parallel with active hemolysis. It is necessary to investigate the real incidence and evolution time of hyposplenism in these patients, because their risk factors for thromboembolism are inconsistent between studies and there is no solid evidence to recommend extended thromboprophylaxis [6]. The peripheral blood smear can be a clue.
